# Communication, Feeding and Swallowing Disorders in Neurological Diseases

**DOI:** 10.1155/2022/9851424

**Published:** 2022-04-21

**Authors:** Grigorios Nasios, Lambros Messinis, Efthimios Dardiotis, Jan Kassubek

**Affiliations:** ^1^Department of Speech & Language Therapy, School of Health Sciences, University of Ioannin, Ioannina, Greece; ^2^Laboratory of Cognitive Neuroscience, School of Psychology, Aristotle University of Thessaloniki, Greece; ^3^Department of Neurology, University of Thessaly, Larissa, Greece; ^4^Department of Neurology, University of Ulm, Ulm, Germany

The multifaceted clinical conditions due to neurological diseases seriously burden patients, their families, and caregivers with disabilities and handicaps, including communication and eating-swallowing disorders which are difficult to cope with and have both a high prevalence and substantial impact on quality of life (QoL). Although these deficits affect millions of patients all over the world, their impact and management has not yet been fully investigated and appreciated in clinical settings. This underestimation of communication and eating-swallowing disorders in neurological diseases led us to organize the current special issue. Our aim was to collect relevant studies from international scientific groups and to provoke a discussion about the unmet needs of many sufferers and the heavy duty of scientists working mainly in clinical settings responsible for their management and relief.

Cerebrovascular diseases represent one of the most common causes of morbidity and mortality worldwide [1]. Although in recent years the rates of stroke in wealthy countries have declined, it has increased to double figures in underdeveloped countries, in which, furthermore, the rates of stroke-related disability and mortality are at least 10 times higher [2]. Among the consequences of stroke, aphasia is one of the most devastating, but also frequent sequelae, present in 21-38% of acute stroke patients and associated with worse outcome and expenditure [3, 4]. On the other hand, poststroke dysphagia, although very common and highly correlated with medical complications, such as aspiration pneumonia, is often difficult to be detected, prevented, and treated, especially in clinical settings without specialized personnel [5–7].

Traumatic brain injuries (TBI) represent a common cause of death and disability (the most common among young adults in developed countries) [8]. Cognitive impairment is frequent and disabling, and depending on its severity, usually a long-term consequence of TBI, responsible for the reduced quality of life and difficulties in many aspects of everyday functioning [9]. Oropharyngeal dysphagia is also common in TBI patients, being reported in almost one-third, 27-30%, of patients [10].

These disorders are also very demanding for people living with progressive neurodegenerative diseases, since functional communication and the ability of self-feeding are globally affected, and their maintenance is highly challenging. Different disorders of language and communication are associated with different forms of dementia, forming distinct clinical profiles and expanding from onset and through the entire course of the underlying diseases, and their evaluation can inform and improve clinical judgments, diagnostically and therapeutically [11]. They include aphasia, apraxia of speech, dysprosodia, alterations in facial expression and gestures, and dysarthria and are present, even early, in most types of dementia [11, 12]. Additionally various eating and swallowing disorders occur in all dementias, such as decrease or increase of appetite, metabolic dysregulation, changes in eating habits, eating apraxia, agnosia of food, and swallowing difficulties [10, 13]. These features appear in the totality of sufferers, during the disease course, and dramatically affect these people's lives [13]. It is of specific concern that many of these disorders share common neural pathways and mechanisms and can occur simultaneously in the same patient.

Cognitive disorders, mainly affecting executive functions, memory, and attention systems, and emotions interfere with functional communication and food consumption, forming a complex and therapeutically challenging landscape [14]. Despite the significant impact of these disorders, in many countries and perhaps most clinical settings, their screening, assessment, diagnosis, and management are not a routine clinical practice yet. The consequences are suboptimal care, earlier loss of independence, reduced quality of life, additional load and costs for care givers, unnecessary gastrointestinal tubing, and serious complications.

With pharmaceutical remedies for the management of such disorders in acquired brain injuries still absent, the importance of cognitive rehabilitation and multidisciplinary approaches, including, but not limited to, neurologists, clinical neuropsychologists, neuro-radiologists, ENT, and specialized speech-language pathologists, was highlighted by us, in a dedicated special issue hosted in this journal [15].

In the current special issue, we host six novel articles from international groups of colleagues. Four of them are clinical studies, one case report, and one review article.

A. M. Georgiou and M. Kambanaros investigated the effectiveness of two different transcranial magnetic stimulation (TMS) paradigms as treatment options for recovery of language deficits in six persons living with chronic poststroke aphasia, in addition to speech and language therapy. Both paradigms used inhibitory stimulation trajectories, targeting the contralesional hemisphere, and more specifically the right pars Triangularis. Three of them were treated with low frequency, 1 Hz, repetitive TMS, and the remaining three were treated with high frequency, continuous theta burst stimulation. When language abilities and QoL were assessed at baseline, immediately after and two months posttreatment, all subjects showed “trends towards improvement” in several language skills, while the rated QoL showed mixed results. One subject followed for two years sustained the treatment-mediated improvement in comprehension and reading skills over time. The authors discuss possible mechanisms of action of their approach, concluding that inhibitory TMS has the potential to drive neuroplastic changes that facilitate language recovery in their sample.

From the same department, D. Kranou-Economidou and M. Kambanaros presented an instructive case study of a 63-year-old male with poststroke aphasia whose working memory (WM) impairment was targeted by the application of intermittent theta burst stimulation (iTBS) combined with computerized WM training to improve receptive and expressive language skills, and through this, communication efficiency and QoL. The treatment program involved iTBS application to the left dorsolateral prefrontal cortex (DLPFC), an area responsible for WM, for 10 consecutive sessions. The participant received a 3-minute iTBS application followed by 30-minute computer-assisted WM training. Treatment beneficially effected naming, reading, WM, reasoning, narrative, communication efficiency, and QoL. Even though this effectiveness was shown in a single case, the implication of cognitive rehabilitation and TMS, in a subject with poststroke aphasia, traditionally treated only with speech and language therapy, evokes discussion about new horizons in aphasia management.

S. Xu et al. addressed the exciting issue of the association and correlation of recovery of poststroke aphasia (PSA) with upper extremity (UE) motor dysfunction, in other words, whether the presence of PSA affects UE motor performance, and if language function associates with UE motor performance. In this multicenter, cross-sectional study, the UE motor status of 435 stroke patients was compared and correlated with the occurrence of PSA in three periods (1-3 months, 4-6 months, and >6 months). Fugl-Meyer assessment for the upper extremity and action research and arm test were used to evaluate the UE motor status, while Western Aphasia Battery-Aphasia Quotient and Boston Diagnostic Aphasia Examination were used to assess the presence, type, and severity of aphasia. The authors concluded that stroke patients with PSA had worse UE motor performance, while UE motor status and language function showed positive correlations, in which spontaneous speech ability significantly accounted for the associations.

Y. Yu et al. set the question about the relationship between alcohol consumption and cognitive impairment after ischemic stroke, investigating the associations of aldehyde dehydrogenase 2 (*ALDH2*) polymorphisms and alcohol consumption with cognitive impairment in 180 Han Chinese ischemic stroke patients from four community health centers in Bengbu, China. Cognitive status was screened in these patients by using the Montreal Cognitive Assessment (MoCA), and *ALDH2* genotypes were determined using polymerase chain reaction and direct sequencing. The results showed that *ALDH2* polymorphisms (and more specifically, the *ALDH2*∗*2* mutant allele) and higher alcohol consumption had a significant synergistic effect on cognitive impairment and swallowing ability after ischemic stroke, indicating that it may be a useful biomarker for cognitive rehabilitation following ischemic stroke. The authors, furthermore, suggested that *ALDH2* might be involved in the pathogenesis and progression of cognitive impairment after ischemic stroke, as well as having a role in alcohol metabolism.

Regarding feeding disorders and other patient populations, our colleagues M. E. Widman-Valencia et al. studied the relationship between oral motor treatment and the improvement of abilities for feeding and swallowing in boys and girls with cerebral palsy (CP), the most common cause of severe physical disability and motor function deterioration in children. Thirty children between 3 and 14 years old, residing in the state of Yucatán, Mexico, with a diagnosis of CP (predominant diagnosis was spastic CP and tetraplegia), with gross motor function levels from II to V, of whom 50% received oral motor treatment, were included in the study. Results showed significant improvement in mandibular mobility, tongue activity, abnormal reflexes, control of breathing, and general oral motor skills, for those treated, regardless of the severity of gross motor involvement. On the contrary, within the sample that did not receive oral motor treatment, 46% presented low or very low weight, and 40% referred recurrent respiratory diseases. It was concluded that feeding skills significantly improve with treatment. Furthermore, oral motor treatment was associated with a lower presence of respiratory diseases and nutritional compromise.

Finally, C. Petsani and colleagues reviewed the literature on available studies about the efficacy of rTMS in the treatment of different neurodegenerative parkinsonian disorders, namely, progressive supranuclear palsy (PSP), corticobasal degeneration (CBD), multiple system atrophy (MSA), and dementia with Lewy bodies (LBD). These syndromes present with heterogeneous clinical phenotypes, reflecting different underlying pathophysiological mechanisms, being either “synucleinopathies” or “tauopathies,” and are characterized by a variety of motor and nonmotor signs and a lack of disease modifying therapies. For PSP patients, cerebellar rTMS holds promise in tackling postural stability and speech impairment, while rTMS over the motor areas could perhaps also be beneficial for their motor symptoms and LF-rTMS over the right DLPFC for resistant major depression. For MSA patients, targeting the left primary motor cortex (M1) with high frequency (HF) rTMS could have a significant improvement on motor dysfunction, while rTMS over the cerebellum, acting on the abnormal cerebellar-cortical inhibitory neuronal connections, could also prove beneficial. For CBD and LBD, there are still very limited data. Overall, research is still at a preliminary phase, with large, double-blind studies lacking in the literature.

From the contributing articles in this special issue, only a limited proportion of the wide range of communication and feeding disorders that constitute part of the clinical entity of many neurological disorders is presented. Still, we hope that the special issue provides an increased awareness on this topic and the significance of ongoing research related to the combinations of therapies in the associated conditions. The articles may assist clinicians when formulating evidence-based neurorehabilitative interventions and further highlight the necessity of multidisciplinary action and collaboration. However, the need for large-scale multidisciplinary and multisite studies to target the clinical presentation and cooccurrence of communication and eating disorders (e.g., dysarthria and dysphagia in parkinsonian syndromes, multiple sclerosis, or stroke), screening and evaluation tools for assessment of these conditions, and combined neurobehavioral, neuromodulational, and pharmacological interventions for the management of communication and feeding/eating disorders remain a priority.
